# The PedsQL™ Oral Health Scale: feasibility, reliability and validity of the Brazilian Portuguese version

**DOI:** 10.1186/1477-7525-10-42

**Published:** 2012-04-24

**Authors:** Cristiane B Bendo, Saul M Paiva, Claudia M Viegas, Miriam P Vale, James W Varni

**Affiliations:** 1 Department of Pediatric Dentistry and Orthodontics, Faculty of Dentistry, Federal University of Minas Gerais, Av. Antônio Carlos 6627, Belo Horizonte, MG, 31270-901, Brazil; 2 Department of Pediatrics, College of Medicine, Department of Landscape Architecture and Urban Planning, College of Architecture, Texas A&M University, 3137 TAMU, College Station, TX, 77843-3137, USA

**Keywords:** Oral health, PedsQL, Quality of life, Validation, Child, Adolescent

## Abstract

**Background:**

Oral and orofacial problems may cause a profound impact on children’s oral health-related quality of life (OHRQoL) because of symptoms associated with these conditions that may influence the physical, psychological and social aspects of their daily life. The OHRQoL questionnaires found in the literature are very specific and are not able to measure the impact of oral health on general health domains. Consequently, the objective of this study was to evaluate the psychometric properties of the Portuguese version for Brazilian translation of the Pediatric Quality of Life Inventory™ (PedsQL™) Oral Health Scale in combination with the PedsQL™ 4.0 Generic Core Scales.

**Methods:**

The PedsQL™ Oral Health Scale was forward-backward translated and cross-culturally adapted for the Brazilian Portuguese language. In order to assess the feasibility, reliability and validity of the Brazilian version of the instrument, a study was carried out in Belo Horizonte with 208 children and adolescents between 2 and 18 years-of-age and their parents. Clinical evaluation of dental caries, socioeconomic information and the Brazilian versions of the PedsQL™ Oral Health Scale, PedsQL™ 4.0 Generic Core Scales, Child Perceptions Questionnaire (CPQ_11-14_ and CPQ_8-10_) and Parental-Caregiver Perception Questionnaire (P-CPQ) were administered. Statistical analysis included feasibility (missing values), confirmatory factor analysis (CFA), internal consistency reliability, and test-retest intraclass correlation coefficients (ICC) of the PedsQL™ Oral Health Scale.

**Results:**

There were no missing data for both child self-report and parent proxy-report on the Brazilian version of the PedsQL™ Oral Health Scale. The CFA showed that the five items of child self-report and parent proxy-report loaded on a single construct. The Cronbach's alpha coefficients for child/adolescent and parent oral health instruments were 0.65 and 0.59, respectively. The test-retest reliability (ICC) for child self-report and parent proxy-report were 0.90 [95% confidence interval (CI) = 0.86-0.93] and 0.86 (95%CI = 0.81-0.90), respectively. The PedsQL™ Oral Health Scale demonstrated acceptable construct validity, convergent validity and discriminant validity.

**Conclusions:**

These results supported the feasibility, reliability and validity of the Brazilian version of the PedsQL™ Oral Health Scale for child self-report for ages 5–18 years-old and parent proxy-report for ages 2–18 years-old children.

## Background

Health-related quality of life (HRQOL) is a multidimensional concept, consisting at the minimum of the physical, psychological (including emotional and cognitive), and social health dimensions delineated by the World Health Organization [1,2]. A generic HRQOL instrument enables comparisons across diverse pediatric populations, including chronic health conditions, as well as benchmarking with healthy populations [3,4].

While traditionally there has been a tendency to treat the oral cavity as a reference point anatomically independent from the rest of the individual’s body, oral health is an integrant part of overall health and contributes significantly to general well-being [5]. Oral diseases are the most common chronic diseases in the childhood, mainly dental caries [6,7]. They are considered of high public health importance because of their prevalence, their deleterious impact on patient overall health, and the costs of treatment [6]. Children who have dental caries may experience a profound negative impact on their oral health-related quality of life (OHRQoL) because of oral symptoms associated with this condition, as well as potential influence on psychological and social aspects of their daily life [7-11]. The hypothesis that the severity of dental caries is associated with the negative impact on OHRQoL has been supported by several studies [11-14].

The OHRQoL questionnaires found in the literature are very specific and are not able to measure the impact of oral health on general health domains [15]. To fill this gap in the literature, the Pediatric Quality of Life Inventory™ (PedsQL™) Oral Health Scale was developed in the United States (U.S.) to measure children’s general oral health status in the evaluation of children and adolescents as a component of general HRQOL [16]. The PedsQL™ Oral Health Scale was designed to be used in conjunction with the PedsQL™ 4.0 Generic Core Scales to provide an overall measure of OHRQoL [16].

The objective of the present study was to evaluate the psychometric properties of the PedsQL™ Oral Health Scale, which was translated and cross-culturally adapted to the Portuguese language for Brazil.

## Methods

### Study area and design

The study was carried out in Belo Horizonte, capital of the state of Minas Gerais, located in the central southeastern region of Brazil. The study was conducted in 2011 with children and adolescents between 2 and 18 years-old and their parents. A total of 243 families of children/adolescents and their parents participated in the study, 35 of which were participants in the cognitive interviewing translation phase of the study, consisting of the adaptation and translation of the PedsQL™ Oral Health Scale for Brazilian Portuguese language, with the remaining 208 families participating in the field test phase to assess the feasibility, reliability and validity of the Brazilian version of the PedsQL™ Oral Health Scale.

### PedsQL™ Oral Health Scale

The PedsQL™ Oral Health Scale was developed in the United States as a generic measure of pediatric OHRQoL [16]. It was designed to be used in conjunction with the PedsQL™ 4.0 Generic Core Scales [16]. The Oral Health Scale is composed of five items and has two parallel instruments for child self-report and parent proxy-report. Child and adolescent self-report includes ages 5–7, 8–12, and 13–18 years. Parent proxy-report includes ages 2–4 (toddler), 5–7 (young child), 8–12 (child), and 13–18 (adolescent), and assesses parent’s perceptions of their child’s oral health. The items for each of the forms are essentially identical, differing in developmentally appropriate language, or first or third person tense. A 5-point response scale is utilized across child, adolescent, and parent proxy-report (0 = never a problem; 1 = almost never a problem; 2 = sometimes a problem; 3 = often a problem; 4 = almost always a problem). To further increase the ease of use for the young child self-report (ages 5–7), the response scale is reworded and simplified to a 3-point scale (0 = not at all a problem; 2 = sometimes a problem; 4 = a lot of a problem), with each response choice anchored to a happy to sad faces scale [4,16]. Items are reverse-scored and linearly transformed to a 0–100 scale (0 = 100, 1 = 75, 2 = 50, 3 = 25, 4 = 0), so that higher scores indicate better OHRQoL. Scale Scores are computed as the sum of the items divided by the number of items answered (this accounts for missing data). If more than 50% of the items in the scale are missing, the Scale score is not computed [17].

### Other measures

The Brazilian version of PedsQL™ 4.0 Generic Core Scales was administered with the PedsQL™ Oral Health Scale [18]. The PedsQL™ 4.0 Generic Core Scales are multidimensional child self-report and parent proxy-report scales developed as the generic core measure to be integrated with the PedsQL™ Disease-Specific Modules for measuring HRQOL in children and adolescents ages 2 to 18. The Generic Core Scales consists of 23 items divided into four scales: Physical Functioning Scale (8 items), Emotional Functioning Scale (5 items), Social Functioning Scale (5 items), and School Functioning Scale (5 items). As in the PedsQL™ Oral Health Scale, it has the same response options and includes child self-report form for ages 5 to 7, 8 to 12, and 13 to 18. Parent proxy-report form includes ages 2 to 4 (toddler), 5 to 7 (young child), 8 to 12 (child), and 13 to 18 (adolescent) [4,18]. This instrument was adapted cross-culturally and validated for use in Brazilian children/adolescents and their parents, exhibiting satisfactory psychometric properties for the Brazilian Portuguese version [18]. Higher scores indicate better generic HRQOL.

The Brazilian versions of the Child Perceptions Questionnaire (CPQ_11-14_ and CPQ_8-10_) [10,19] was answered by children/adolescents between 8 and 14 years of age, as well as all participant parents answered the Brazilian version of the Parental-Caregivers Perception Questionnaire (P-CPQ) [20]. Only items of the oral domain of each instrument were collected. The questions refer only to the frequency of events in the previous three months. The items have 5 rated response options: ‘never’ = 0, ‘once or twice’ = 1, ‘sometimes’ = 2, ‘often’ = 3 and ‘every day or almost every day’ = 4. A ‘don’t know’ response was permitted for P-CPQ and scored as 0. Higher scores for these instruments indicate worse OHRQoL.

Additionally, the parents completed a form that contained social, demographic and economic questions. This form also has questions about their child general and oral health.

The children/adolescents were examined to diagnose dental caries, and rated according to the Decayed, Missing and Filled Teeth Index (DMFT) [21]. Dental clinical examinations were performed at school during daytime hours. One pediatric dentist (CBB) examined all children/adolescents using a disposable mouth mirror (PRISMA®, São Paulo, SP, Brazil). All teeth were examined for the diagnosis of dental caries. The examiner used appropriate equipment to protect against individual cross-infection, with all necessary instruments and materials packaged and sterilized in sufficient quantities for each workday. The examiner participated in a training and calibration exercise. Twenty children/adolescents were randomly selected and included in the calibration process. They were re-examined after a 15 days interval for the calculation of intra-examiner agreement. Cohen’s Kappa value was 0.98, thereby demonstrating excellent intra-rater agreement.

### Adaptation and translation of the PedsQL™ Oral Health Scale

Translation and cross-cultural adaptation of the PedsQL™ Oral Health Scale into the Portuguese language for Brazil followed international guidelines for instrument linguistic validation [22,23]. In order to generate the Brazilian Portuguese version of the PedsQL™ Oral Health Scale, two bilingual translators whose native language was Brazilian Portuguese performed independently the translation from the original English-language PedsQL™ Oral Health Scale. The two translated versions were analyzed by a committee composed of dental specialists to discuss the translations and reached agreement on a single reconciled version. This version of the questionnaire was backward translated by a professional, bilingual translator native English speaker. This translator did not have access to the original instrument. A comparison between the original version and the backward translated version was performed [15,18,22-24]. The translated instruments were administered to a sample of 35 families (15 children/adolescents between 5 and 18 years of age and 20 parents of children/adolescents between 2 and 18 years of age) to determine whether the translation (instructions, items and response choices) was acceptable; whether it was understood in the way it is supposed to be; and whether the language used was simple and appropriate. The Respondent Debriefing technique was used for the cognitive interviews. Five children/adolescents and parents in each age range group (2–4, 5–7, 8–12, 13–18 years) were interviewed. Some modifications in the translated version of the PedsQL™ Oral Health Scale were made after cognitive interviews as suggested by children/adolescents and their parents.

### Assessment of feasibility, reliability and validity of the Brazilian version of the PedsQL™ Oral Health Scale

Two hundred and eight (208) families of children/adolescents and their parents participated in the field test phase of the study. The children’s age range was between 2 and 17 years and the average age was 7.96 [standard deviations (SD) = 4.50]. The majority of the sample was composed of girls (58.7%), whose race/ethnicity was white or brown (49.0% and 43.5%, respectively). The main respondent of the parent proxy-report instruments was mothers (83.2%), with more than 8 years of education (76.3%) and higher income (61.4%). The mean DMFT index of children/adolescents was 0.99 (SD = 1.90). The children/adolescents’ frequency of tooth brushing was related as three times a day by 42.5% of parents. The majority of parents considered their child/adolescent’s oral health as very good or good (73.8%) (Table 1).

**Table 1:** Sociodemographic Characteristics

Variables	N (%)
Child age (mean ± SD)	7.96 (4.5)
Child gender	
Male	86 (41.3)
Female	122 (58.7)
Child race/ethnicity	
White	98 (49.0)
Brown	87 (43.5)
Black	11 (5.5)
Other	4 (2.0)
Parents’ level of education	
≤ 8 years of study	49 (23.7)
> 8 years of study	158 (76.3)
Family income	
≤ 3 minimum wage	76 (38.6)
> 3 minimum wage	121 (61.4)
Social vulnerability Index of child’s home (mean ± SD)	0.39 (0.16)
Relationship of the respondent with the child	
Mother	173 (83.2)
Father	27 (13.0)
Other	8 (3,8)
Decayed, missing and filled teeth index (mean ± SD)	0.99 (1.90)
Tooth brushing	
Once a day or less	16 (7.7)
Twice a day	83 (40.1)
Three times a day	88 (42.5)
Four times a day or more	20 (9.7)
Dental floss	
Yes	110 (53.1)
No	97 (46.9)
Child’s general health reported by parents	
Very good/good	190 (92.7)
Fair/Poor/very poor	15 (7.3)
Child’s oral health reported by parents	
Very good/good	152 (73.8)
Fair/Poor/very poor	54 (26.2)

Children/adolescents were recruited from public schools in the city of Belo Horizonte, Brazil. The Brazilian version of the PedsQL™ Oral Health Scale was self-administered to children/adolescents between 8 and 18 years-old and parents. Children between 5 and 7 years of age were interviewed by one interviewer (CBB). The participants also answered the Brazilian version of the PedsQL™ 4.0 Generic Core Scales and the Brazilian versions of the CPQ_11-14_, CPQ_8-10_ and P-CPQ. On the same day as the questionnaire administration, the children/adolescents were examined to diagnose any dental caries. Two weeks later, the participants were asked to answer the Brazilian version of PedsQL™ Oral Health Scale again.

### Ethical considerations

Following authorization from the Human Research Ethics Committee of the Federal University of Minas Gerais (Brazil), permission was granted by the administration of the each school selected. A letter of invitation was then sent to the children/adolescents and their parents, explaining the aim, characteristics, importance and methods of the study and asking for their participation.

### Statistical analyses

The Statistical Package for the Social Sciences (SPSS for Windows, version 19.0, SPSS Inc., Chicago, IL, USA) was used for the majority of the data analyses. Data analysis included descriptive statistics (frequency distribution, ceiling and floor effects, means and SD). Acceptable floor or ceiling effects are less than or equal to 15% [25]. Feasibility was determined from the percentage of missing values.

To evaluate the construct validity and confirm the single dimension of the Brazilian version of the PedsQL™ Oral Health Scale, a confirmatory factor analysis (CFA) was conducted. The LISREL for Windows program (version 8.8, Scientific Software International Inc., Lincolnwood, IL, USA) was used for this analysis. To unstandardized solutions, the pattern of fixed and free factor loadings was held constant. The chi-square (*X*^2^) statistic is an extremely sensitive statistical test, not interpretable in a standardized way and not a practical test of model fit [26,27]. Thus, consistent with recommendations in the literature [26,28,29], several different model indices of practical fit were evaluated including the Normed Fit Index (NFI), Comparative Fit Index (CFI), Goodness of Fit Index (GFI), Adjusted Goodness of Fit Index (AGFI) and Root Mean Squared Error of Approximation (RMSEA). For the NFI, CFI, GFI and AGFI indices, excellent model fit is suggested by values greater than or equal to 0.95, while acceptable model fit is suggested by values between 0.90 and 0.95. Excellent model fit is suggested by RMSEA values less than or equal to 0.06, while acceptable model fit is suggested by RMSEA values between 0.06 and 0.08 [15,30].

The test-retest and internal consistency reliability of the Brazilian version of the PedsQL™ Oral Health Scale was determined. The internal consistency reliability was estimated using Cronbach's Alpha (α) Coefficient. Values ≥ 0.70 are considered acceptable for comparisons between groups [4,15,24,31,32]. The test-retest reliability was assessed using the Intraclass Correlation Coefficient (ICC). The ICC was measured according to the following values: ≤0.40 weak correlation; 0.41– 0.60 moderate correlation; 0.61–0.80 good correlation; and 0.81–1.00 excellent correlation [15,24,33].

The construct validity was determined by calculation of the intercorrelations among the Brazilian versions of the PedsQL™ Oral Health Scale and PedsQL™ 4.0 Generic Core Scales using Pearson correlation coefficients [15,16,34-36]. Computing the intercorrelations among scales provides initial information on the construct validity of an instrument [37]. We hypothesized greater oral health-specific symptoms or problems would correlate with lower overall generic HRQOL based on the conceptualization of disease-specific symptoms as causal indicators of generic HRQOL [38]. Pearson Product Moment Correlation coefficients are designated as small (0.10-0.29), medium (0.30-0.49), and large (>0.50) [39]. Correlational analysis was also conducted to assess the convergent validity between child self-report scores of the Brazilian version of the PedsQL™ Oral Health Scale and the Brazilian versions of CPQ_11-14_ and CPQ_8-10_, as well as between parent-proxy report scores of the Brazilian version of the PedsQL™ Oral Health Scale and the Brazilian versions of P-CPQ [16].

The known-groups method was used to establish the discriminant validity by comparisons of means and SD between groups diagnosed with and without dental caries experience and parents perception of child’s oral health. This analysis was conducted using Independent Samples t-tests.

The ICC was used to measure the agreement between child self-report and parent proxy-report for the Brazilian versions of the PedsQL™ Oral Health Scale and PedsQL™ 4.0 Generic Core Scales [15,16].

## Results

### Descriptive analysis

There were no missing data for both child self-report and parent proxy-report of the Brazilian version of the PedsQL™ Oral Health Scale. A low rate of missing data occurred for child self-report and parent proxy-report of the Brazilian version of PedsQL™ 4.0 Generic Core Scales, 0.2% and 0.8%, respectively.

Regarding the Brazilian version of PedsQL™ 4.0 Generic Core Scales, children/adolescents and parents had similar mean scores for the total scale (81.16, SD = 13.14; 80.49, SD = 12.55, respectively). However, parents reported a higher mean score for the Brazilian version of PedsQL™ Oral Health Scale (87.75, SD = 14.71) than children/adolescents (83.34, SD = 16.63) (Table 2).

**Table 2:** Descriptive analyses and Cronbach’s coefficient alpha (α) for the Brazilian versions of the PedsQL™ 4.0 Generic Core Scales and PedsQL™ Oral Health Scale

Scale	Number of items	Mean	SD	Percent floor	Percent ceiling	Cronbach’s α
**Child self-report**						
Total Generic Core Scale	23	81.16	13.14	0	4.1	0.85
Physical Functioning	8	84.28	14.31	0	13.8	0.68
Emotional Functioning	5	72.31	21.53	0	14.5	0.70
Social Functioning	5	88.64	15.10	0	39.3	0.65
School Functioning	5	77.48	19.08	0.7	11.7	0.68
Oral Health Scale	5	83.34	16.63	0	22.8	0.65
**Parent proxy-report**						
Total Generic Core Scale	23	80.49	12.55	0	1.4	0.85
Physical Functioning	8	85.20	16.26	0.5	26.4	0.77
Emotional Functioning	5	70.05	17.24	0	4.3	0.68
Social Functioning	5	87.56	15.04	0	37.0	0.74
School Functioning	5	75.29	18.19	0	11.6	0.76
Oral Health Scale	5	87.15	14.71	0	37.5	0.59

There were no floor effects for the child self-report and parent proxy-report of the Brazilian versions of the PedsQL™ Oral Health Scale and PedsQL™ 4.0 Generic Core Scales. Ceiling effects were detected for both instruments (Table 2).

### Confirmatory factor analysis

**Figure 1:**
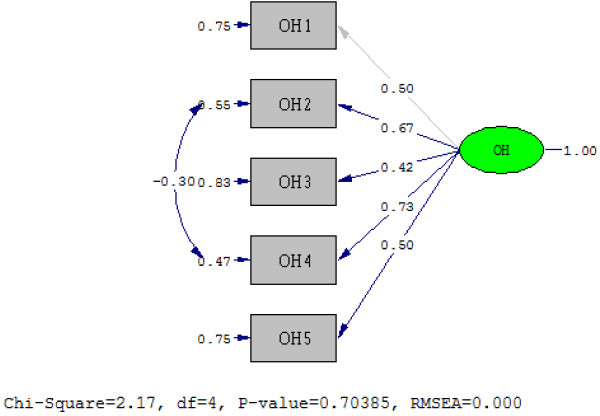
Confirmatory factor analysis on the 5-item of the Brazilian version of the PedsQL™ Oral Health Scale child self-report.

**Figure 2:**
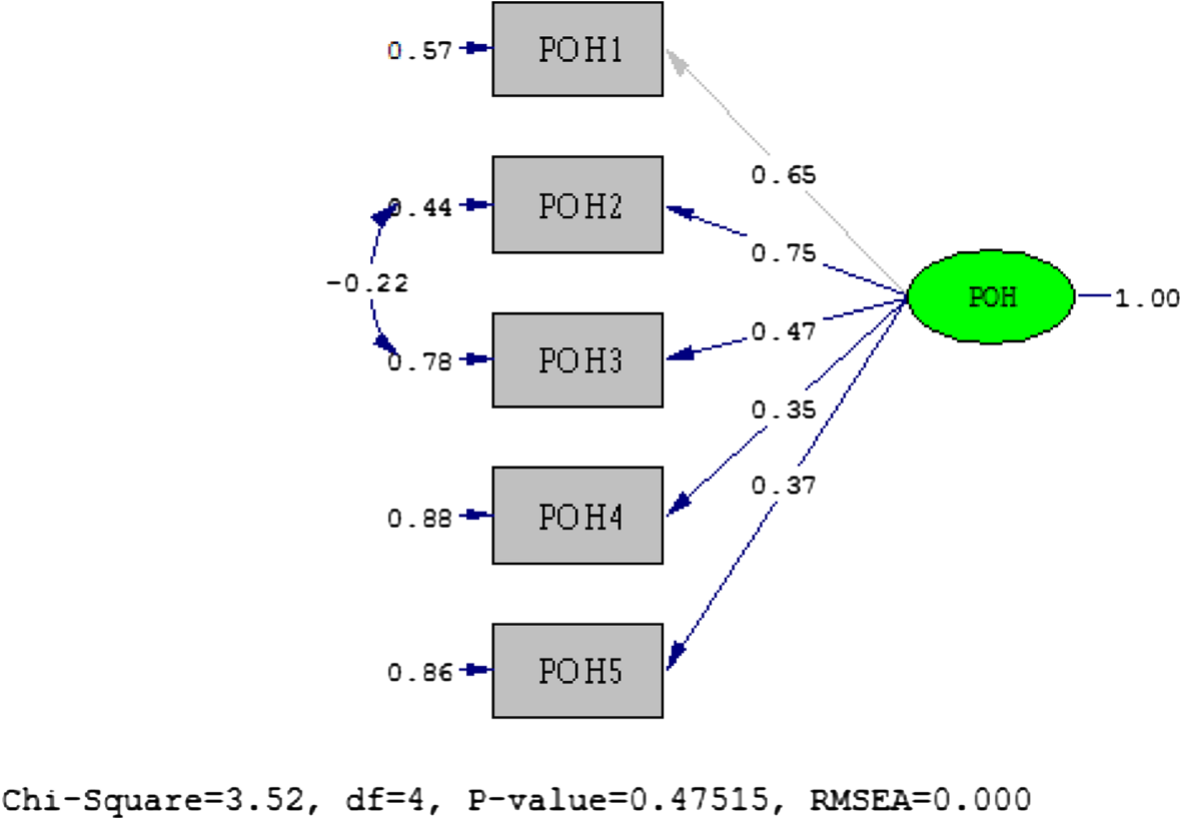
Confirmatory factor analysis on the 5-item of the Brazilian version of the PedsQL™ Oral Health Scale parent proxy-report.

In order to confirm the unidimensionality of the Brazilian version of the PedsQL™ Oral Health Scale, a CFA was conducted. The results show that the five items of both the child self-report and parent proxy-report versions loaded on a single latent variable. However, the first CFA for both child and parent models did not fit the data well. For child self-report, the goodness of fit statistics was *X*^2^ = 12.69, degrees of freedom (df) = 5, p-value = 0.026, NFI = 0.90, CFI = 0.93, GFI = 0.97, AGFI = 0.90 and RMSEA = 0.097. For parent proxy-report, the fit statistics was *X*^2^ = 12.56, df = 5, p-value = 0.028, NFI = 0.91, CFI = 0.94, GFI = 0.98, AGFI = 0.93 and RMSEA = 0.082. In order to improve the overall model fit, an error covariance between OH2 (Having tooth pain when eating or drinking something hot, cold, or sweet) and OH4 (Having gum pain) was added for child self-report; and between POH2 (Having tooth pain when eating or drinking something hot, cold, or sweet) and POH3 (Having teeth that are dark in color) for parent proxy-report. The modified models are showed in Figures 1 and 2. The goodness of fit indices for modified child self-report model was *X*^2^ = 2.18, df = 4, p-value = 0.70, NFI = 0.98, CFI = 1.00, GFI = 0.99, AGFI = 0.98 and RMSEA = 0.0. The indices for the modified parent proxy-report model was *X*^2^ = 3.57, df = 4, p-value = 0.47, NFI = 0.98, CFI = 1.00, GFI = 0.99, AGFI = 0.97 and RMSEA = 0.0.

### Reliability

Table 2 displays the internal consistency reliability values estimated by Cronbach's alpha coefficient. The Cronbach's alpha for the child self-report and parent proxy-report total scale scores of the Brazilian versions of the PedsQL™ 4.0 Generic Core Scales were both 0.85. The majority of scales of the Brazilian versions of the PedsQL™ 4.0 Generic Core Scales showed values around 0.70, considered acceptable. The Cronbach's Alpha Coefficient for the Brazilian versions of the PedsQL™ Oral Health Scale were less than 0.70, with a child self-report alpha of 0.65 and a parent proxy-report alpha of 0.59. Test-retest reliability as measured by ICC showed an excellent correlation for child self-report and parent proxy-report of the Brazilian version of PedsQL™ Oral Health Scale across the two weeks of the test-retest interval (ICC = 0.90, 95%CI = 0.86-0.93; ICC = 0.86, 95%CI = 0.81-0.90, respectively).

### Validity

The intercorrelations among the Brazilian versions of the PedsQL™ Oral Health Scale and PedsQL™ 4.0 Generic Core Scales (total scale score and scale scores) were calculated by Pearson correlation in order to determine construct validity and are showed in Table 3. Almost all correlations have a significance level at 0.01 and can be classified as medium or large. For child self-report, the Brazilian version of the PedsQL™ Oral Health Scale was more strongly correlated with the Emotional Functioning Scale (r = 0.56, p < 0.01). Regarding parent proxy-report, the Generic Core Scales Total Scale score was most correlated with the Brazilian version of the PedsQL™ Oral Health Scale (r = 0.45, p < 0.01).

**Table 3:** Intercorrelations among Brazilian versions of the PedsQL™ 4.0 Generic Core Scales and PedsQL™ Oral Health Scale for child self-report and parent proxy-report

**Scale**	**TGC**	**PF**	**EF**	**SF**	**SchF**	**OH**
TGC	--	0.82**	0.78**	0.70**	0.75**	0.51**
PF	0.85**	--	0.49**	0.52**	0.42**	0.36**
EF	0.71**	0.47**	--	0.35*	0.49**	0.56**
SF	0.70**	0.47**	0.33**	--	0.42**	0.19*
SchF	0.70**	0.42**	0.33**	0.43**	--	0.40**
OH	0.45**	0.41**	0.38**	0.28**	0.22**	--

TGC: Total Generic Core Scales; PF: Physical Functioning Subscale; EF: Emotional Functioning Subscale; SF: Social Functioning Subscale; SchF: School Functioning Subscale; OH: Oral Health Scale;

Intercorrelations for child self-report are presented above the diagonal, and intercorrelations for parent proxy-report are presented below the diagonal. Categories of the correlations are small (0.10), medium (0.30) and large (0.50).

* P < 0.05; ** P < 0.01.

Pearson correlations were conducted between the Brazilian versions of the PedsQL™ Oral Health Scale and the oral symptoms domain of the Child Oral Health Quality of Life (COHQoL) questionnaires (CPQ_11-14_, CPQ_8-10_, P-CPQ). The CPQ_11-14_ (6 items) was answered by adolescents with age range of 11 to 14 years; the CPQ_8-10_ (5 items) was responded by children 8 to 10 years of age. The results demonstrated an inverse correlation among child self-report of the CPQ_11-14_ and CPQ_8-10_ questionnaires and Brazilian version of the PedsQL™ Oral Health Scale (r = −0.61, p < 0.01; r = −0.86, p < 0.01; respectively). The P-CPQ (6 items) was answered by all of the 208 parents, and their scores were inversely correlated with parent proxy-report of the Brazilian version of the PedsQL™ Oral Health Scale (r = −0.58, p < 0.01).

Table 4 shows the known-groups analysis to support discriminant validity by the comparisons between groups known to different in oral health status. For this analysis, five outliers who did not have dental caries on examination but who still reported very low scores (worse symptoms) on the Brazilian version of the PedsQL™ Oral Health Scale, were removed from the analyses since they skewed the data excessively. As predicted, the mean score of the child self-report form of the Brazilian version of the PedsQL™ Oral Health Scale was statistically significant higher for children who did not experience dental problems (DMFT = 0) than those with dental problems (DMFT **≥** 1) (p = 0.043). The similar result was found for parent proxy-report (p < 0.001). Moreover, both child self-report and parent proxy-report of the Brazilian version of the PedsQL™ Oral Health Scale was able to discriminate among groups based on parent's perceptions of child’s oral health (p = 0.001, p < 0.001; respectively).

**Table 4:** Comparison of the Brazilian versions of the PedsQL™ 4.0 Generic Core Scales and PedsQL™ Oral Health Scale scores for Decayed, Missing and Filled Teeth Index (DMFT) categories and Parents Perception of Child’s Oral Health

**Scale**	**DMFT**		**p-value**	**Parents perception of child’s oral health**		**p-value**
	**0**	**≥1**		**Very good/good**	**Fair/poor/very poor**	
**Child self-report**						
Total Generic Core Scale	82.0(13.4)	81.2(11.5)	0.709	83.4(11.8)	77.4(13.7)	**0.010**
Physical Functioning	85.1(14.7)	83.4(13.6)	0.486	85.5(13.7)	81.5(15.2)	0.128
Emotional Functioning	72.5(21.0)	73.5(21.4)	0.796	75.8(20.4)	66.8(20.1)	**0.019**
Social Functioning	88.0(16.3)	90.2(12.6)	0.391	89.3(14.5)	87.9(15.9)	0.609
School Functioning	79.6(17.7)	77.3(17.6)	0.440	81.8(14.8)	71.0(21.6)	**0.005**
Oral Health Scale	87.1(11.9)	81.8(17.1)	**0.043**	87.9(12.4)	77.4(16.7)	**0.001**
**Parent proxy-report**						
Total Generic Core Scale	82.7(12.2)	77.0(12.4)	**0.002**	83.2(10.9)	73.0(13.8)	**<0.001**
Physical Functioning	88.1(15.2)	80.3(17.2)	**0.002**	87.9(14.1)	77.6(19.9)	**0.001**
Emotional Functioning	72.5(16.5)	66.2(18.1)	**0.014**	72.8(15.6)	62.6(20.0)	**0.002**
Social Functioning	86.8(14.2)	87.9(15.3)	0.627	89.4(13.6)	81.9(17.4)	**0.007**
School Functioning	77.4(16.5)	72.5(20.1)	0.079	78.9(16.2)	66.6(19.9)	**<0.001**
Oral Health Scale	90.6(11.5)	81.6(15.9)	**<0.001**	91.2(10.7)	76.8(17.0)	**<0.001**

Independent Samples *T*-Test;

Results in bold type significant at 5% level;

DMFT: decayed, missing and filled teeth index.

### Parent–child agreement

There was good agreement between the children/adolescents and their parents on the Brazilian version of the PedsQL™ Oral Health Scale, with a value of 0.74. The ICC revealed a moderate agreement between children/adolescents and parents for the total scale score and three of the four scale scores of the Brazilian version of the PedsQL™ 4.0 Generic Core Scales. The Emotional Functioning Scale (0.61) demonstrated good agreement among children/adolescents and their parents (Table 5).

**Table 5:** Intraclass correlations between child self-report and parent proxy-report and for the Brazilian versions of the PedsQL™ 4.0 Generic Core Scales and PedsQL™ Oral Health Scale

**Scale**	**ICC**
Total Generic Core Scale	0.54
Physical Functioning	0.50
Emotional Functioning	0.61
Social Functioning	0.34
School Functioning	0.45
Oral Health Scale	0.74

ICC: Intraclass correlation coefficients.

ICCs < 0.40 show poor to fair agreement, 0.41–0.60 moderate agreement, 0.61–0.80 good agreement and >0.80 excellent agreement.

All with P < 0.01.

## Discussion

The present study demonstrated that the translated and cross-cultural adapted version of the PedsQL™ Oral Health Scale was reliable and valid for Brazilian children/adolescents and their parents. This instrument is useful to capture a child’s general oral health status, and was designed to be used in conjunction with PedsQL™ Generic Core Scales [16].

There were minimal missing responses on the PedsQL™ Generic Core Scales and no missing responses on the PedsQL™ Oral Health Scale. These results suggest that children/adolescents and parents understood the items and were able to provide data with a good quality regarding the child’s generic HRQOL and OHRQoL. The means scores of the PedsQL™ Oral Health Scale and PedsQL™ Generic Core Scales were high, indicating good health on average. These findings were expected given that the sample was from a general public school population, in which generic HRQOL and oral health would be expected to be in the normal range. The low mean for the DMFT found in the present sample is consistent with the sample characteristics of primarily healthy schoolchildren. Our results about dental caries experience (DMFT) are supported by several studies that showed a decrease of dental caries experience in Brazilian children and adolescents [40-42]. We found higher (better) scores for both the PedsQL™ 4.0 Generic Core Scales and the PedsQL™ Oral Health Scale then found in an Iranian sample on the Persian translation validation of the PedsQL™ Oral Health Scale into their culture [15]. The Iranian sample showed a mean of DMFT of 2.45 (SD = 2.66), while our study found DMFT of 0.99 (SD = 1.90). The low rate of dental caries experience in these Brazilian schoolchildren may also explain the ceiling effects detected in our study. As the majority of our sample was composed of healthy children and adolescents, with a low DMFT average, it was expected that the scores of the PedsQL™ Oral Health and PedsQL™ Generic Core Scales would be high, which indicate good OHRQoL and HRQOL, respectively.

The CFA demonstrated that the five items of the Brazilian version of the PedsQL™ Oral Health Scale measure the same unidimensional construct for child self-report and parent proxy-report forms, which corroborate with what was proposed in the development of the instrument in the U.S. [16]. The validation study to develop the Iranian version of PedsQL™ Oral Health Scale also concluded that the instrument was unidimensional based on their CFA findings [15]. For the PedsQL™ Oral Health child-self-report version, items OH2 and OH4 appear to have something in common, sharing a common measurement error term. For the parent proxy-report version, a covariance was added between POH2 and POH3 errors. The Iranian validation study of the PedsQL™ Oral Health Scale also proposed sharing measurement errors among variables for parent proxy-report in order to improve the model fit [15]. Measures of goodness of fit in CFA analysis summarize the discrepancy between observed values and the values expected under the hypothetical model. Poor fit can be remedied by adding a covariance between errors that reflect all other sources of variance in the items not explained by the construct (Brazilian version of the PedsQL™ Oral Health Scale). A measurement error correlation reflects the assumption that two items measure something in common that is unique to them and not represented in the model. These follow-up procedures modify certain model parameters in order to improve the overall model fit. If adding the covariance between errors is based on the modification indices and is theoretically meaningful, as well as does not result in an unidentified model, it can be helpful by decreasing the model’s overall Chi-square, and consequently improve the overall fit of the model [30].

The internal consistency reliability for the PedsQL™ Oral Health Scale was lower than acceptable values. These values are not in accordance with the Iranian versions of PedsQL™ Oral Health Scales; this previous study found greater Cronbach’s alpha values [15]. On the other hand, the U.S. validation study found a low Cronbach’s alpha for child self-report and affirmed that measures with fewer items tend to be more sensitive to this kind of analysis [16]. However, our results are consistent with the findings of the CPQ_11-14_, CPQ_8-10_ and P-CPQ validation studies for Brazil. These studies obtained values of Cronbach’s alpha < 0.70 for their oral symptoms domains (CPQ_11-14_ = 0.52, CPQ_8-10_ = 0.63, P-CPQ = 0.44) [10,19,20]. Further research on the Brazilian translation of these instruments is indicated in samples with high dental caries and dental disease in order to ascertain whether the low Cronbach’s alpha is sample specific or a reflection of a generally orally healthy sample. Cronbach’s alpha may also have been attenuated by the low variability in the Oral Health Scale items for this sample since this was a generally healthy schoolchildren population who reported few oral health problems [32]. Consequently, the utilization of the PedsQL™ Oral Health Scale in pediatric patient with known oral health problems is strongly indicated in order to more fully test Cronbach’s alpha in this scale.

Additionally, in discussing the low Cronbach’s alpha values, the authors of the U.S. initial validation study suggested alternative methods for assessing measurement consistency, such as test-retest reliability [16]. In contract to the Cronbach’s alpha findings, the test-retest reliability for the Brazilian version of the PedsQL™ Oral Health Scale was excellent across time. Given that this was primarily a healthy schoolchildren sample, the observed stability in oral health across a two week interval would be expected for a reliable measure. The ICC values found in our study are higher than those from the Iranian study [15], which may reflect that their sample had greater dental problems. Thus, in our primarily healthy sample, the Brazilian version of the PedsQL™ Oral Health Scale was highly reproducible.

The present study provided evidence for the construct validity of the Brazilian version of the PedsQL™ Oral Health Scale. This Oral Health Scale was statistically correlated with the Brazilian version of the PedsQL™ Generic Core Scales for the total scale score and the scale scores. This finding is in accordance with the previous PedsQL™ Oral Health Scale validated studies conducted in the U.S. and Iran [15,16]. Utilizing intercorrelations between the PedsQL™ Generic Core Scales and other PedsQL™ Scales has been useful for supporting construct validity in a number of studies [15,16,34-36].

The convergent validity of the PedsQL™ Oral Health Scale was supported by statistically significant correlations between the Brazilian version of the PedsQL™ Oral Health Scale and the Brazilian versions of the COHQoL questionnaires (CPQ_11-14_, CPQ_8-10_ and P-CPQ). The inverse correlation occurred because higher scores in the PedsQL™ Oral Health Scale indicates better OHRQoL, while higher scores in COHQoL questionnaires indicates worse OHRQoL. Only the oral symptoms domain of each COHQoL questionnaire was used for these correlational analyses, in accordance with the U.S. validation study of the PedsQL™ Oral Health Scale, which found similar results for the correlations between these two instruments [16].

The PedsQL™ Oral Health Scale was able to discriminate between groups with and without dental caries problems (DMFT **≥** 1 and DMFT = 0, respectively). As expected, the mean score for the PedsQL™ Oral Health Scale was lower for children/adolescents who experienced dental problems than for those who never had this experience. This result corroborates the findings of the Iran validation study of the PedsQL™ Oral Health Scale [15] and other OHRQoL studies [10,11] that children/adolescents with dental problems manifest worse OHRQoL. The five outliers removed from the database for this analysis were generating errors and distortions in statistical analysis since even though they did not have dental caries on examination they still reported very low scores (worse symptoms) on the Brazilian version of the PedsQL™ Oral Health Scale. We found that parents’ perception of child oral health predicted child self-report and parent proxy-report OHRQoL scores. Similar results were found by correlational analysis in previous studies [10,19,20]. It is interesting to note that children diagnosed with dental caries and whose parents reported worse child’s oral health had statistically significant worse HRQOL for parent proxy-report of the PedsQL™ Generic Core Scale for Total Scale, Physical Functioning and Emotional Functioning than their counterparts. The same results did not occur for child self-report, since there was not found any difference in generic HRQOL between DMFT groups, and only children whose parents reported worse child’s oral health had statistically significant lower scores for the PedsQL™ Generic Core Scales Total Scale Score, Emotional Functioning and School Functioning Scale scores. Similar findings were reported in the Iran validation study of the PedsQL™ Oral Health Scale, which found no association between DMFT and the PedsQL™ Generic Core Scales for child self-report [15].

As the hypothesis that oral health problems can negatively impact the child’s HRQOL, mainly in parents views, was supported by the know-group analysis, it is important to include a measure of oral health when measuring generic HRQOL. Oral health problems which can result from specific diseases or medical treatments for chronic diseases can place children at a greater risk for developing overall health problems. Oral mucositis have been shown to occur in patients undergoing chemotherapeutic management for cancer [43]. Individuals who are HIV + present a higher risk of periodontal diseases, dental cavities, xerostomy, candidiasis and Kaposi’s sarcoma [44,45], in addition to more severe impact on OHRQoL [46]. Additionally, periodontal disease could be a risk factor for coronary heart disease [47]. Consequently, it is evident that general and oral health should be considered jointly [7,16,46].

Children and parents showed a moderate agreement in the majority of the scales of the Brazilian version of the PedsQL™ Generic Core Scales, with the Social Functioning Scale presenting poor to fair agreement. These results corroborate the findings with the U.S and Iranian studies, which found the worst value for ICC in Social Functioning Scale [15,16]. Regarding the Brazilian version of the PedsQL™ Oral Health Scale, the agreement between children and parents was good, corroborating the results of the Iranian study [15]. The U.S. study found a lower agreement, being in the moderate agreement range [16]. It is not common to find good agreement between children and parents perceptions regarding HRQOL. The high level of agreement on the PedsQL™ Oral Health Scale may be explained because the items asked about observable occurrences, and it was potentially easier for parents and children to have the same perception given the item content [15,16].

This study has some limitations that must be recognized. The clinical examinations were held in schools, which did not permit the use of X-rays. Thus, dental problems were obtained by visual dental examination only by a pediatric dentist. This procedure may underestimate the presence of dental caries. However, the sample of schoolchildren allowed for various degrees of dental problems on the DMFT, including children free of dental problems. Since the PedsQL™ Oral Health Scale was designed to measure children`s general oral health status in the evaluation of children and adolescents HRQOL at the general population level as well as in the clinic setting, this sample was deemed appropriate for the initial validation study in Brazil. Finally, other more serious oral conditions that may potentially significantly impact children’s OHRQoL were not assessed, such as malocclusion and traumatic dental injury. Another possible limitation was that the study was conducted only in public schools, resulting in a homogeneous socioeconomic sample, which may minimize the generalization of the results.

## Conclusions

In summary, the results of the present study support the feasibility, reliability and validity of the Brazilian Portuguese version of the PedsQL™ Oral Health Scale for 5–18 years-old child self-report and parent proxy-report of 2–18 years-old children. Given the low Cronbach’s alpha and ceiling effects, it is recommended that the Brazilian version of the PedsQL™ Oral Health Scale be tested in dental clinic samples, including children with more severe dental problems as the next steps of the validation process in Brazilian children and adolescents. Finally, item revisions of the instrument may be in order to improve the psychometric properties pending further validation in clinical samples with chronic health conditions.

## Abbreviations

OHRQoL = Oral health-related quality of life; PedsQL™ = Pediatric Quality of life Inventory™; CPQ = Child Perceptions Questionnaire; P-CPQ = Parental-Caregiver Perception Questionnaire; ICC = Intraclass correlation coefficients; CI = Confidence interval; CFA = Confirmatory factor analysis; HRQOL = Health-related quality of life; U.S = United States; DMFT = Decayed, missing and filled teeth index; SPSS = Statistical Package for the Social Sciences; α = Alpha; NFI = Normed fit index; CFI = Comparative fit index; GFI = Goodness of fit index; AGFI = Adjusted goodness of fit index; RMSEA = Root mean squared error of approximation; SD = Standard deviations; COHQoL = Child Oral Health Quality of Life; OH1 = Having tooth pain; OH2 = Having tooth pain when eating or drinking something hot, cold, or sweet; OH3 = Having teeth that are dark in color; OH4 = Having gum pain; OH5 = Having blood on his or her toothbrush after brushing; X2 = Chi-square; Df = Degrees of freedom.

## Competing interests

Dr. Varni holds the copyright and the trademark for the PedsQL™ and receives financial compensation from the Mapi Research Trust, which is a nonprofit research institute that charges distribution fees to for-profit companies that use the Pediatric Quality of Life Inventory™.

## Authors’ contributions

CBB, CMV, SMP, MPV and JWV conducted the literature review, conceptualized the rationale and designed the study; CBB and CMV performed the data collection; CBB, SMP and JWV performed the statistical analysis and interpretation of the data. CBB, CMV and JWV drafted the manuscript. All authors read and approved the final manuscript.
